# Country-Level Assessment of Missed Opportunities for Vaccination in South Africa: Protocol for Multilevel Analysis

**DOI:** 10.2196/16672

**Published:** 2020-09-28

**Authors:** Duduzile Ndwandwe, Ntombenhle J Ngcobo, Abdu A Adamu, Chukwudi Nnaji, Thandiwe Mashunye, Arlette M Leufak, Sara Cooper, Olalekan A Uthman, Charles S Wiysonge

**Affiliations:** 1 Cochrane South Africa South African Medical Research Council Cape Town South Africa; 2 Centre for Evidence-based Health Care, Division of Epidemiology and Biostatistics Department of Global Health, Faculty of Medicine and Health Sciences Stellenbosch University Cape Town South Africa; 3 School of Public Health and Family Medicine University of Cape Town Cape Town South Africa; 4 Warwick-Centre for Applied Health Research and Delivery Division of Health Sciences University of Warwick Medical School Coventry United Kingdom

**Keywords:** South Africa, vaccination coverage, missed opportunities for vaccination, implementation science

## Abstract

**Background:**

Vaccination is one of the greatest public health interventions of all time. Vaccination coverage in South Africa has shown a steady improvement in reaching the national target. However, while there is progress nationally, there are districts within the country that are below the set target for vaccination coverage. One of the main drivers of suboptimal vaccination coverage is thought to be missed opportunities for vaccination.

**Objective:**

This study aims to understand the magnitude and determinants of missed opportunities for vaccination in South Africa.

**Methods:**

The 2016 South African Demographic and Health Survey will be used to conduct multilevel regression analyses to determine individual and contextual factors associated with missed opportunities for vaccination in South Africa. The perspectives of parents attending health care facilities in South Africa will be explored through exit interviews and focus group discussions. Similarly, perspectives of the health care providers will be sought to understand enablers and barriers to vaccination coverage at the facility level. Insights to such factors will aid in designing tailor-made interventions to improve vaccination coverage in South Africa.

**Results:**

Ethical review submission is planned for October 2020. Data collection is expected to be underway in January 2021.

**Conclusions:**

The extent of missed opportunities in South Africa coupled with the associated factors presents an opportunity for efforts to increase uptake in districts where vaccination coverage is below the national target. Population-level data such as those from the 2016 South African Demographic Health Survey will provide an idea of the magnitude of missed opportunities for vaccination in South Africa at the national and subnational levels. The findings of the study will inform national and subnational policy implementation on vaccinations and help to find context-specific interventions to improve vaccination coverage.

**International Registered Report Identifier (IRRID):**

PRR1-10.2196/16672

## Introduction

### Background

Vaccinations currently save between 2 and 3 million children every year [1]. However, vaccines can only prevent childhood diseases if they reach the intended target populations [2]. In low- and middle-income countries, the benefits of vaccinations, which can be direct and indirect, are not being maximized for children, as routine immunization remains suboptimal [2]. Immunization coverage (with the third dose of diphtheria-tetanus-pertussis–containing vaccine; DTP3) had reached 90% by 2018 in 129 countries, the majority of which were high-income countries [3]. According to the World Health Organization (WHO), the 10 countries with the most unvaccinated children are Angola, Brazil, the Democratic Republic of the Congo, Ethiopia, India, Indonesia, Nigeria, Pakistan, the Philippines, and Viet Nam [3]. Immunization coverage is one of the key measures for assessing the performance of national immunization programs [4]. DTP3 is commonly used as a surrogate indicator for immunization [5]. Low- and middle-income countries are dependent on the Expanded Program on Immunization framework to ensure that all children receive their recommended vaccinations [6]. The Expanded Program on Immunization is supported by local health ministries to provide vaccinations to infants at no cost [4].

However, there are challenges related to accessing the Expanded Program on Immunization. These include accessing far-to-reach health care facilities, vaccine stock-outs in the facilities, and more importantly, missed opportunities for vaccination and vaccination hesitancy [7,8]. In addition to the challenges associated with the Expanded Program on Immunization, another important issue that affects vaccination coverage is poor data collection and reporting. Vaccination coverage data quality was highlighted in 2012 pointing to multiple issues surrounding data collection [9,10], compilation, and transfer in generating immunization data. Countries need to collect and report high-quality vaccination data to enable assessment of immunization performance. In turn, delivery of the vaccines to populations in need can be improve [6].

In South Africa, there has been substantial progress in immunization coverage, access to free health care, and prevention of malnutrition and mother-to-child transmission of HIV. However, the current levels of under-5 mortality in the South Africa are still very high indicating that the United Nation Sustainable Development Goals' target of less than 25 deaths per 1000 live births by 2030 may not be reached [11]. The top causes of under-5 mortality are neonatal conditions, diarrhea, pneumonia, and HIV-related infections [12]. Multiple efforts are being made to improve immunization coverage; there is a need for the improvement of health service delivery vis-a-vis establishing synergy between health programs to reduce missed opportunities for vaccination [10].

Missed opportunities for vaccination refer to any contact with health services by persons who are eligible for vaccination (eg, unvaccinated or partially vaccinated and free of contraindications to vaccination), which does not result in them receiving one or more of the vaccinations for which they are eligible [7]. Missed opportunities for vaccination occur in two major settings: (1) during visits for vaccination and other preventive services and (2) during visits for curative services. In both settings, eliminating missed opportunities will increase the overall immunization coverage in the population and thus prevent vaccine-preventable diseases.

Recommended strategies for reducing missed opportunities for vaccination emphasize the usefulness of periodic monitoring to evaluate the quality of vaccination program performance at the health service level as well as evaluating progress toward reducing missed opportunities [13].

The Expanded Program on Immunization is one of the most successful and cost-effective public health initiatives to reduce infant morbidity and mortality from vaccine-preventable diseases. The benefits of immunization are so immense that in 2011, the World Health Assembly put forth a resolution to declare a Decade of Vaccines (2011-2020) [4]. Furthermore, target 3.8 of the Sustainable Development Goals emphasizes the importance of immunization and, as such, calls for “access to safe, effective, quality and affordable medicines and vaccines for all” by 2030 [14]. Improved coverage of early childhood immunization is essential to achieving Sustainable Development Goals goal 3.8. Gavi, the Vaccine Alliance is calling for a universally applicable vaccine indicator to “reach and sustain 90% national coverage and 80% in every district with all vaccines in national programs” to be one of the measures of Sustainable Development Goal 3.8 [15].

Additionally, further research is needed to assess various factors attributed to missed opportunities in different districts across the country. The proposed study will provide novel insights on the magnitude and determinants of missed opportunities for vaccination to enable tailor-made strategies for addressing various factors unique to different districts, thus filling the key knowledge gaps, which is important for achieving vaccination goals in South Africa. Given the pragmatic nature of the project, its involvement of key national vaccination stakeholders and provincial and district stakeholders will generate knowledge that has the potential to inform policy decisions at the national level and which can easily be implemented at provincial and district levels. 

### Aims and Objectives

The objectives of the study are as follows: (1) to examine the influence of individual-, neighborhood- and province-level socioeconomic factors on missed opportunities for vaccination in South Africa; (2) to explore reasons for missed opportunities for vaccination from the perspectives of caregivers of children aged 0-23 months attending primary health care facilities in Western and Eastern Cape provinces of South Africa; (3) to explore reasons for missed opportunities for vaccination from the perspectives of health care providers in primary health care facilities in Western and Eastern Cape provinces of South Africa.

## Methods

### Preliminary Identification of Factors

We will use the 2016 South African Demographic and Health Survey (SADHS). This will be a cross-sectional study. Briefly, the SADHS is a nationally representative household survey conducted in South Africa which uses a multistage, stratified sampling design with households as the sampling unit. Within each sample household, women and men meeting the eligibility criteria are interviewed. The survey findings represent the full target population because the samples are not self-weighting, and therefore, account for unequal selection probabilities as well as nonresponses [16]. SADHS is composed of a household questionnaire, a women’s questionnaire, and in most countries, a men’s questionnaire. We will use the WHO’s definition of missed opportunities for vaccination as the outcome variable, defined as a binary variable that takes the value of 1 if the child 12-23 months had any contact with health services but remained unvaccinated to any vaccination dose for which the child is eligible. Contact with health services will be defined using the following 6 variables: skilled birth attendance, baby postnatal check within 2 months, received vitamin A dose in first 2 months after delivery, had health care and medical treatment of diarrhea, fever, or cough. We will limit the analysis to one child per woman to minimize the overrepresentation of women with more than one child in the age category. Individual-level factors will be included in the models: child's age, sex of the child (male versus female), high birth order (>4 birth order), child’s birth weight, number of children under the age of 5 years in the household, maternal age completed years (15 to 24, 25 to 34, 35 or older), employment status (working or not working), maternal education (no education, primary, or secondary or higher), and media access (radio, television, internet, or newspaper). SADHS does not collect direct information on household income and expenditures. We will use the SADHS wealth index as a proxy indicator for socioeconomic status. The methods used in calculating the SADHS wealth index have been described elsewhere [17,18].

An index of economic status for each household will be constructed using principal components analysis based on the following household variables: number of rooms per house and ownership of a car, motorcycle, bicycle, fridge, television, and telephone, as well as any kind of heating device. From these criteria, the SADHS wealth index quintiles (poorest, poorer, middle, richer, and richest) will be calculated and used in the subsequent modeling. Clustering within the same geographical living environment will be described as a neighborhood. Neighborhoods will be based on sharing a common primary sample unit within the SADHS data [19,20]. We will consider neighborhood socioeconomic disadvantage for the community-level variable in this study. Neighborhood socioeconomic disadvantage will be operationalized with a principal component comprised of the proportion of respondents with no education (illiterate), unemployed, rural resident, and living below the poverty level (asset index below 20% poorest quintile). A standardized score with mean 0 and standard deviation 1 will be generated from this index; with higher scores indicative of lower socioeconomic position. We will divide the resultant scores into 5 quintiles to allow for nonlinear effects and provide results that are more readily interpretable in the policy arena.

### Participant Sampling

We purposively selected the Western Cape and Eastern Cape provinces for the study to represent the urban and rural settings. The minimum sample required is estimated at 620 in the two provinces which will include both the parents and health care providers This sample size will be used for quantitative surveys and focus group discussion data collection. While the sample size has been estimated, this type of analysis allows reiteration to ensure that saturation is obtained. The estimation is based on the following assumptions: a prevalence of missed opportunities for vaccination of 32.2%, from a previous study [21]; an acceptable margin of error of 5%; nonresponse rate of 20%; and a design effect of 1.5 [21-23]. Design effect will be considered to account for clustering since respondents are embedded within specific health facilities [22-24]. For feasibility and logistical reasons, 10 primary health care facilities in each of the 2 districts—OR Tambo District in the Eastern Cape Province and Cape Town Metropolitan Municipality in Western Cape Province—will be randomly selected by cluster sampling technique. Each selected primary health care facility will be considered as a cluster. From each selected primary health care facility, all eligible and consenting caregivers with a child aged 0-23 months will be included.

For the focus group discussions, a purposively selected group of parents of children aged 0-23 months attending the selected facilities will be sampled from the participating primary health care facilities, based on findings from the quantitative analyses to incorporate diversity in terms of socioeconomic status, race, and class. Each group will comprise of 6 to 10 mothers. Selected parents (or caregivers) will be homogenous in terms of place of residence which allows the views by class, socioeconomic status, and level of education. In each province, at least 3 focus group discussions will be conducted. If necessary, additional sessions will be held until data saturation is reached.

For the in-depth interviews, we will purposively select 10 health facility staff, including vaccinators, clinical staff, and facility managers. Participants will be selected by taking into consideration roles, gender, and geographic location.

### Participant Recruitment

Before participant recruitment, all necessary approvals from the regulatory and provincial health departments will be obtained. Participants will be recruited in the selected primary health care clusters with the assistance of the health care workers in those facilities. We will recruit parents (or caregivers) bringing their children for vaccinations to take part in the exit interviews. All participants will be asked to consent to participating by signing an informed consent form. Once participants enrolled in the study complete their exit interviews, appointments for the focus group discussions will be organized.

Previous research in South Africa [25,26] has identified that the vast majority of missed opportunities for vaccinations are caused by health facility obstacles, and thus health care workers will also provide informed consent to participate in the study to understand service provider-perspective on missed opportunities for vaccination and collaboratively find interventions that are most suited for those settings.

### Data Collection

#### Exit Interviews

Face-to-face interviews will be conducted for both parents (or caregivers) and health care providers. For parents, quantitative data will be collected through face-to-face exit interviews using an interviewer-administered structured questionnaire. This questionnaire is an adaptation of a WHO tool for assessing missed opportunities for vaccination in health care settings [27]. The exit interview questionnaire that will be used in this study has 6 sections: (1) data on the child; (2) data on the child's caregiver (or mother); (3) use of vaccination card and information on vaccination administered; (4) today's vaccination; (5) quality of the vaccination service; (6) reasons for getting vaccinated. Exit interviews will be conducted by trained (male and female) staff, who are fluent in both English and the South African Language most spoken in the study area. They will administer the structured questionnaire using mobile tablets. Pilot testing of the questionnaire will be conducted in a separate local area to ensure clarity and suitability of the questions. Data will be collected using REDCap (Vanderbilt University) mobile app on mobile tablets. Data quality assurance will be done using key/value pairs. Data collected will be stored on a secured database. After quantitative data collection, the data file will then be exported from REDCap to STATA (version 14.1; StataCorp LLC) for analysis. For the qualitative data, we will use NVivo (version 1.0; QSR International) to assist with data management and analysis. All personal identifiers will be removed from the interview and discussion transcripts before analysis. All recordings will be deleted upon completion of the study.

#### In-depth Interviews

Semistructured interviews will allow for in-depth exploration of the contextual factors and mechanisms of missed opportunities for vaccination from the experiences and perspectives of health workers and facility managers in the selected primary health care facilities.

All sessions will be conducted in private rooms within the selected health facilities, at a convenient time for the participants. The interview process will be open-ended and flexible, allowing participants the freedom to develop and deeply express their responses.

#### Focus Group Discussions

A qualitative research design will be used to explore the reasons for missed opportunities for vaccination from the perspectives of parents (or caregivers) [28]. Focus group discussion will be conducted face-to-face in a private room within the health facilities, at a convenient time for the participants. Discussions will last approximately one hour each. Each participant will be allowed to contribute during discussion and will maintain a circular sitting arrangement. A semistructured question guide will be used during the discussion. Participants will be asked about their experiences, and perception regarding vaccination, vaccination services and missed opportunities for vaccination. This question guide will be piloted in a primary health facility in South Africa to ensure clarity and suitability of questions.

### Data Management and Analysis

#### Quantitative Data Management and Analysis

For SADHS data, descriptive analysis will be used to describe the distribution of respondents by key variables. Multivariable logistic multilevel regression models will be used to analyze the association between individual compositional and contextual factors associated with missed opportunities for vaccination. We will specify a 3-level model for binary response reporting missed opportunity for vaccination or not, for a child (at level 1), in a neighborhood (at level 2), living in a province (at level 3). Models will be constructed as (1) an empty or unconditional model without any explanatory variables, specified to decompose the amount of variance that exists between province and neighborhood levels; (2) containing only individual-level factors; (3) containing only neighborhood-level factors; (4) containing only province-level factors; and (5) simultaneously controlled for individual-, neighborhood- and province-level factors (full model).

For quantitative data from exit-interviews, missed opportunities for vaccination will be calculated using child's date of birth and date at which the last vaccination doses were administered. This will then be compared to the standard due date to determine if a missed opportunity has occurred. Children who are fully immunized for age will be categorized as *no missed opportunities for vaccination* while those who are not fully immunized for age will be categorized as *missed opportunities for vaccination*. Explanatory variables will be grouped into 3 levels as follows: (1) child-related factors (age of child, sex of the child, birth order); (2) caregiver-related factors (relationship with a child, marital status, level of education, occupation, mode of transport to the health facility, duration of transport to the health facility, exposure to media); and (3) health facility–related factors (refusal to offer vaccination, checking of vaccination card, charged fee for vaccination, charged fees for vaccination card, location characteristics, type of health facility, number of health workers, number of vaccinators). The distribution of explanatory variables (child, parent, and facility-related factors) by the outcome (missed opportunity for vaccination) will be calculated. We will specify a 2-level model for binary response reporting missed opportunity for vaccination or not with child and parent (or caregiver) as level 1, both nested within primary health care facilities (as level 2). Models will be constructed as (1) an empty (null) model with no explanatory variable; (2) containing only individual-level (child and caregiver) factors; (3) containing only facility-level factors; and (4) simultaneously controlled for child and caregiver-related and facility-level factors (full model).

The results of fixed effects (measures of association) will be reported as odds ratios with 95% credible intervals—95% CrIs. This Bayesian statistical inference approach provides probability distributions for measures of association, which can be summarized with 95% credible intervals, rather than 95% confidence intervals. A 95% credible interval can be interpreted as a 95% probability that the parameter takes a value in the specified range.

The possible contextual effects will be measured by the intraclass correlation and median odds ratio [29,30]. We will measure the similarity between respondents in the same neighborhood and within the same province using intraclass correlation. The intraclass correlation represents the percentage of the total variance in the probability of missed opportunities for vaccination that is related to the neighborhood- and province-level (ie, measure of clustering of odds of missed opportunities for vaccination in the same neighborhood and province). The median odds ratio measures the second- or third-level (neighborhood or province) variance as odds ratios and estimates the probability of missed opportunities for vaccination that can be attributed to neighborhood and provincial context. A median odds ratio equal to one indicates no neighborhood or province variance. Conversely, the higher the median odds ratio, the more important the contextual effects are for understanding the probability of missed opportunities for vaccination. We will check for multicollinearity among explanatory variables by examining the variance inflation factor [31], all diagonal elements in the variance-covariance matrix for correlations between –1 and 1, and diagonal elements for any elements close to 0.

MLwinN software (version 3.0; University of Bristol) will be used for the analyses [32]. Parameters will be estimated using the Markov chain Monte Carlo procedure [32]. The Bayesian deviance information criterion will be used as a measure of how well the different models fitted the data. A lower value on deviance information criterion indicates a better fit of the model [33]. Scatter plots of performance, as a percentage, against the number of missed opportunities for vaccination children (the denominator for the percentage) will be generated. The mean provincial performance and exact binomial 3-sigma limits will be calculated for all possible values for the number of cases and used to create a funnel plot using the method described by Spiegelhalter [34,35]. If a province lies with the 99% CI, it has a crude missed opportunities for vaccination rate that is statistically consistent with the average rate (common-cause variation). If a country lies outside the 99% CI, then it has a crude missed opportunities for vaccination rate that is statistically different from the average rate (special-cause variation).

#### Qualitative Data Management and Analysis

Focus group discussions will be recorded using a portable audiorecorder and transcribed verbatim. Transcription will be done by a professional; however, each transcript will be checked for accuracy by the principal investigator. For the in-depth interviews, the template analysis approach will be used for coding and organizing data segments for analysis [36]. This method allows for flexible thematic analysis as the codebook can be adapted to the context of the study [37]. Two codebooks will be developed. The first codebook will specify factors identified from the discussion. For this codebook, the themes will be identified inductively [38]. In the second codebook, domains of the theoretical domains framework will be specified [39]. This is a validated framework with 14 domains that is useful for identifying barriers in implementation research [39]. The themes identified in the first codebook will be deductively adapted to domains of the theoretical domains’ framework. The coder will identify causes of missed opportunities for vaccination from the interview transcripts, and then map each identified factor to a domain of theoretical domains framework. To avoid overlapping codes, only the most relevant code will be mapped to a particular domain. Coded data will be used for analysis. Analysis summaries for a combination of factors will be used to populate an analytic matrix. Illustrative quotations will be used in analytical summaries.

#### Integrative Analysis (Mixed Methods Approach)

We will conduct a mixed methods approach to integrate the quantitative data collected through the 2016 SADHS and structured questionnaires from exit interviews of caregivers, the qualitative data collected through the focus group discussions of parents (or caregivers) of children aged 0-23 month, and the qualitative data collected from the in-depth interviews of health care providers. Methodological integration considerations will be taken in the design, analysis, and reporting stages of the study. We will adopt the convergent design to better understand the complexity of missed opportunities for vaccination from the perspectives of caregivers and health care providers. The convergent design will compare both quantitative and qualitative data analyzed at the same time followed by an integrative analysis [40]. From the thematic analysis of the qualitative data, themes will be compared with the corresponding variable from questionnaires, and the results will be displayed as qualitative summaries and quotations for each domain-participant combination, thus allowing a full understanding of the complexity surrounding missed opportunities for vaccination from the health care service user [40,41].

### Considerations

Ethical approval will be obtained from the South African Medical Research Council. We will also obtain permission from the appropriate authority of the South African Department of Health. The study process will comply with the requirements of the latest version of the Declaration of Helsinki (7th revision, 2013). Verbal and written information about the study will be provided to all participants taking part in interviews and focus group discussions. For the qualitative data, with permission of participants, all interviews and focus groups will be digitally recorded and subsequently transcribed verbatim. All digital recordings will be erased following transcription, and all identifying information will be removed from transcripts. Participant confidentiality and anonymity will thus be ensured. The consent form will make explicit the following aspects: the voluntary nature of participation, that there will be no negative consequences if they decide not to participate, that they will explicitly be asked for permission for the interview to be digitally recorded, and that this is also voluntary. Written consent will be obtained from all research participants before proceeding with interviews or focus groups.

Details from interviews and focus group discussions will be entered into a study-specific database on the day of collection (stakeholder group, participant ID, etc). Study data, including audio recordings, will be stored on password-protected computers and shared with the study team only. All digital recordings on recorders will be destroyed following safe storage and transcription, and identifying information will be removed from all transcripts. Reports of the findings will not identify individual participants. Participant anonymity and confidentiality will thus be ensured.

No risks to participants or researchers are expected. All potential participants for interviews or focus groups are not considered to be vulnerable individuals or groups. However, participants may be uncomfortable expressing criticisms of vaccination programs. Where there is this potential, and where participants identify concerns, we will reassure participants of the steps that will be taken to ensure confidentiality. For participants in focus groups, we will remind participants at the outset that while the researchers undertake to maintain confidentiality, we cannot guarantee that other focus group participants will. At the start of the focus group, we will discuss the importance of everyone involved maintaining confidentiality after the focus group but will explain that there is an inherent risk of breaches of confidentiality in this method. We will ensure that participants are aware of this risk.

## Results

Ethical review submission is planned for October 2020. Data collection is expected to be underway in January 2021.

## Discussion

### General

Not much is known about the burden and determinants of missed opportunities for vaccination in South Africa. Although a 2015 cross-sectional study conducted across selected health facilities in Cape Town found a low prevalence of 5% for missed opportunities for vaccination among children aged 0 to 5 years, the magnitude of the burden remains uncertain [42]. The study [42] found inadequate immunization training and knowledge among health care providers, as well as heavy workloads, as the main factors associated with missed opportunities to vaccinate children. In South Africa, the value that vaccinations deliver remains far below the substantial benefits that they can offer [43]. While there have been significant investments and efforts in ensuring universal access to immunization services in South Africa, it is evident that in some districts and many neighborhoods, there are eligible children who are missing out on this very critical health intervention [44,45]. How much of this gap is due to missed opportunities for vaccination remains uncertain. The mechanism and contextual factors associated with missed opportunities for vaccination in South Africa are less certain, and therefore, this study seeks to unpack these contextual factors in efforts to improve vaccination coverage in South Africa.

Our multilevel analytical approach considers the hierarchical structure of 2016 SADHS data and will enable us to simultaneously examine the effects of individual and contextual factors on missed opportunities for vaccination. Furthermore, we will use an innovative mixed methods approach to integrate quantitative and qualitative data from the SADHS, exit interviews, focus group discussions, and in-depth interviews, to better understand the complexity of missed opportunities for vaccination from multiple perspectives.

While there is currently limited evidence on the structural and contextual factors responsible for missed opportunities for vaccination in different contexts, research efforts in this area have increased in recent years. As a result, there has been an increasing body of evidence on the prevalence of missed opportunities for vaccination and associated factors over the last decade, including in African and other low- and middle-income countries contexts [46-51]. To date, however, there remain enormous gaps in what is known. Particularly, very little evidence exists on the burden and determinants of missed opportunities for vaccination in South Africa [42]. Much less certain is the evidence on the role and effectiveness of interventions for addressing missed opportunities for vaccination, with very little evidence on these in Africa, and none in South Africa [52].

Understanding the burden and mechanisms of missed opportunities for vaccination is important for policy and practice as it will provide valuable evidence to enable policymakers and facility managers to consider context-appropriate interventions for strengthening immunization programs. It will help inform institutions of context-appropriate and locally responsive immunization strategies and interventions to optimize immunization access and coverage. Therefore, this study will have local and national policy and practice implications in South Africa and Africa, in general. It will inform strategies for ensuring equitable and improved immunization coverage toward the attainment of the WHO Global Vaccine Action Plan targets, Universal Health Coverage agenda, and health-related UN Sustainable Development Goals.

### Dissemination of Findings

The findings of the study will be shared with stakeholder groups, at consultation workshops, and on Cochrane South Africa websites. At the end of the study, a project report will be shared with all stakeholders who took part in interviews, focus groups, and consultation workshops. The findings will also be communicated through academic publications and conferences. Reporting of the qualitative data will adhere to COREQ [53] guidelines.

## Abbreviations

COREQ: Consolidated Criteria for Reporting Qualitative Research
DTP3: third dose of diphtheria-tetanus-pertussis–containing vaccine
HIV: human immunodeficiency virus
PRISMA: Preferred Reporting Items for Systematic Review and Meta-Analysis
PRISMA-P: Preferred Reporting Items for Systematic Review and Meta-Analysis Protocols
SADHS: South African Demographic and Health Survey
WHO: World Health Organization
